# Myocarditis induced by clozapine and COVID-19 infection: a case report

**DOI:** 10.1192/j.eurpsy.2023.2154

**Published:** 2023-07-19

**Authors:** P. Veloso, M. Gomes, R. Faria, D. Matos, F. Pereira

**Affiliations:** 1 Psychiatry, Hospital de Braga, Braga; 2 Neurology, Unidade Local de Saúde do Alto Minho, Viana do Castelo, Portugal

## Abstract

**Introduction:**

Clozapine is the only available treatment for refractory schizophrenia and is rarely associated with the development of myocarditis. Usually, the onset of symptoms occurs within the first month of treatment. The symptoms of myocarditis include fever, flu-like symptoms, fatigue, and dyspnea, symptoms that overlap with the COVID-19 infection. Coronavirus has been associated with cardiovascular complications, including myocarditis. It is not known whether clozapine increases the risk of developing viral myocarditis in patients with COVID-19 infection.

**Objectives:**

Report a case of myocarditis in a patient treated with clozapine, who also had a history of COVID-19 infection.

**Methods:**

Collection of clinical information and review of the literature.

**Results:**

A 24-year-old man was admitted following severe psychotic symptoms that have been developing for the past several months. He presented with disorganized speech and behavior, paranoid delusions, thought alienation, auditory hallucinations, and blunted affect. He had no known medical co-morbidity, but he had tested positive for COVID-19 the month before admission. The lab and imaging tests and the electrocardiogram (EKG) were normal. He was diagnosed with schizophrenia and after treatment failure with three antipsychotics, the patient was started on clozapine, with symptom improvement. Two weeks after clozapine initiation, he started flu-like symptoms, fever, chest pain, and tachycardia. Lab tests showed leukocytosis (12 400 cells/uL), elevated inflammatory markers (C-reactive protein 143,30 mg/L) and cardiac biomarkers (troponin I 12.139 ng/mL, NT-proBNP 9321 pg/ml). The evaluation for viruses, including SARS-CoV-2, was negative. The EKG revealed ST-segment elevations and a transthoracic echocardiogram showed systolic dysfunction (left ventricular ejection fraction was 37%). Cardiac magnetic resonance confirmed severe left ventricular dysfunction and diffuse myocardial edema. The patient’s symptoms resolved following the discontinuation of clozapine and supportive therapies. Troponin and EKG normalized over the following 7 days. By this time, the patient tested positive for COVID-19.

**Conclusions:**

The temporal relationship with the initiation of clozapine supports the diagnosis of clozapine-associated myocarditis. However, the COVID-19 infection may have played a part in the emergence of cardiac alterations. We hypothesize that the co-occurrence of COVID-19 and clozapine treatment may act synergically as both factors increase the risk of developing myocarditis. However, further studies are needed to evaluate the relationship between these factors. While clinicians should stay alert for the risk of clozapine-associated myocarditis, the overall risk is low, and given the effectiveness of clozapine, as well as the absence of other evidence-based treatments, people with refractory schizophrenia should be given a monitored trial of clozapine, regardless of their COVID-19 status.

**Disclosure of Interest:**

None Declared

